# Reducing the impact of penetrating trauma in the UK: a project by young doctors to teach first responder skills to young offenders

**DOI:** 10.1186/1757-7241-21-S1-A3

**Published:** 2013-05-28

**Authors:** N Rhead, C Neary-Bremer, S Jackson, S Rhead, T Conley, DJ Lockey

**Affiliations:** 1 StreetDoctors, Liverpool, UK

## Background

Interpersonal violence is the third leading cause of death within the European Region (as defined by World Health Organisation (WHO)) [1]. To address this concern the World Health Assembly passed a resolution (WHA 49.25)[2] declaring violence to be a public health priority. Furthermore the assembly passed resolution (WHA 56.24)[3] in 2003 urging continued development of a science-based public health approach to prevention of youth violence.

The public health problem is also specifically present in England where in 2006/2007 National Health Service data shows that 5,720 people were admitted to hospital following “assault by sharp object”[4]. Of these 179 were aged under 16 and a further 752 were aged between 16 and 18. Analysing Home Office data Hall and Innes found that the most likely victims of violent crime are young men aged between 16 and 24. The risk of being a victim of violent crime in this cohort is 13.3%, over four times higher than the average risk (3%)[5].

Other features of high risk groups include; trauma recidivism, previous exposure to violence, fear of violence and social relationships with violent peers. Furthermore those young people who have previously been involved in delinquency and are known to the criminal system are significantly more likely to be participants in carrying and using a weapon[6].

## The StreetDoctors project

In Liverpool, UK a novel prevention and intervention training scheme has been established to decrease the morbidity and mortality from interpersonal violence amongst young people. The only public health intervention of its kind globally *StreetDoctors* utilises medical students to provide high risk young people with the skills, knowledge and confidence to manage victims in the minutes immediately following injury, concentrating mainly on haemorrhage control. They do so because young offenders have been identified as a high risk population who often witness penetrating trauma. Half of the young offenders taught by *StreetDoctors* in Liverpool 2011 had witnessed penetrating trauma in the community and 90% felt that learning basic haemorrhage control techniques was a necessary skill for them and their peer group. When comparing fatal and non-fatal stabbings in Edinburgh, Webb et al found that the “the presence of a bystander capable and willing to request emergency medical assistance” had a positive impact upon the chance of survival from a stab injury[7].

By explaining potential long term consequences of penetrating trauma such as colostomies and physical disability it is hoped that *StreetDoctors* has a strong injury prevention message. The main aim of the intervention project is however to create a reservoir of potential healthcare providers who, unlike healthcare professionals will be able to deliver care at the point of injury. This intervention essentially bridges the gap between injury and treatment to limit blood loss and aims to decrease associated morbidity and increase the chance of survival to hospital. *StreetDoctors* uses a careful and competitive recruitment process to build teams of 10-20 volunteer medical students and doctors in each local area. Recruitment is based on applicants’ ability to communicate in an engaging, inspiring and non-judgemental way. Volunteers are trained by certified trainers in haemorrhage control, basic life support and child protection. Experienced volunteers oversee the development of new branches, supporting new volunteers and ensuring a high standard of teaching is delivered.

*StreetDoctors* teaching is clear, simple and interactive. Role plays, DVDs, visual demonstrations and practical skill sessions are utilised to ensure complex information is understood and is easily recalled during a time of high stress. To ensure that familiarity and continuity are encouraged the same volunteers teach a group of 6-10 young people at a local centre on weekday evenings. In the first session basic haemorrhage control is taught. The first session covers topics such as basic anatomy and physiology, short and long term consequences of penetrating trauma, how to call an ambulance and management of bleeding before professional help arrives. The second session a week later targets drug use and is entitled “What to do if someone collapses”. This includes Cardiopulmonary Resuscitation (CPR) and the recovery position following a summary of the previous week’s session. Each session takes approximately 90 minutes.

The teaching which started in 2007 in Liverpool, UK has been delivered to more than 1,500 young people across five cities including London and Manchester. In this short period and with funding of just £3500 *StreetDoctors* is already aware of three cases where teaching has been successfully utilised.

## The future of the project

Penetrating trauma is a major public health concern amongst the majority of UK urban populations. *StreetDoctors* will therefore expand to all major UK cities which have a problem with youth violence within the next five years. Following expansion within the UK *StreetDoctors* believes that the training it provides is applicable to high risk young people globally and therefore expects to establish international collaboration and new projects.

The teaching model used by *StreetDoctors* and the concept of utilising young highly skilled professional students to teach is essential to the success of the training program. *StreetDoctors* believe that this teaching model can be used to develop training programs in other areas of public health where high risk young people are at risk. Of particular concern sexual health and young women’s safety in gang culture and illicit drug use are two areas where *StreetDoctors* hope to develop an intervention in coming years.

Young people who are known to the criminal justice system are understandably a difficult cohort to study particularly when attempting follow up and establishing utilisation of the education provided by the project. Therefore *StreetDoctors* hope to develop a research model to combat this and determine long term impacts of teaching on attitudes and behaviour. In the short term *StreetDoctors* will evaluate skills retention following teaching through the use of survey and Objective Structured Clinical Examination (OSCE) techniques. Finally it is planned that the impact of the teaching upon morbidity/length of hospital stay and mortality will be evaluated.
